# A PC-controlled module system for automatic sample preparation and analysis

**DOI:** 10.1155/S1463924695000034

**Published:** 1995

**Authors:** Östen Einarsson, Lars Hansén

**Affiliations:** 1 Analys Modul AB S191 62 Sollentuna Sweden; 2 National Institute of Occupational Health SOLNA S-171 84 Sweden

## Abstract

A simple automatic analytical system, consisting of separate modules, for liquid chromatography has been constructed. The different parts of the automatic machine are an auto sampler, an auto dispenser, a selector valve with eight channels, a heater/cooler, a mixing chamber and a pressure air driven injector valve. The process was controlled by a PC from an easily changeable run protocol. The system was applied to analysis of primary amines. The analysis was performed as a pre-column derivatization reaction of the amines and separation by isocratic reversed-phase HPLC with fluorescent detection. Reproducibility and analytical precision have been studied. Comparison between automatically and manually made derivatization reaction and injection was also made. The automatic system was easy to handle, cost-effective and gave good reproducibility.


Journal of Automatic Chemistry, Vol. 17, No. 1 (January-February 1995), pp. 21-24

### A PC-controlled module system for automatic sample preparation and analysis

**Östen Einarsson**
*Analys Modul AB, S191 62 Sollentuna, Sweden*

**and Lars Hansén**
*National Institute of Occupational Health, S-171 84 SOLNA, Sweden*

*A simple automatic analytical system, consisting of separate modules, for liquid chromatography has been constructed. The different parts of the automatic machine are an auto sampler, an auto dispenser, a selector valve with eight channels, a heater/cooler, a mixing chamber and a pressure air driven injector valve. The process was controlled by a PC from an easily changeable run protocol. The system was applied to analysis of primary amines. The analysis was performed as a pre-column derivatization reaction of the amines and separation by isocratic reversed-phase HPLC with fluorescent detection. Reproducibility and analytical precision have been studied. Comparison between automatically and manually made derivatization reaction and injection was also made. The automatic system was easy to handle, cost-effective and gave good reproducibility.*

### Introduction

Automatic analytical systems for high-performance liquid chromatography have greatly simplified the analysis of a large number of samples and increased analytical precision. Many sophisticated systems are available, especially for health care routine screening of biological samples. A fully automated system (including sample preparations) is called a robotic automation system and is often very expensive [1]. For laboratory research work, where experimental sample numbers are of moderate size and it needs to be relatively simple to change analytical parameters and detectors, a simpler system is desirable. One way of achieving this is to combine different commercial components with a suitable analytical technique. The whole system should then be controlled from a personal computer.

Flow injection analysis (FIA) instruments are an important group of instruments [2]. Apparatus for this technique can be built up by the combination of commercial instruments. This paper presents an alternative automated flow system that could be called MIA (multiple injection analysis). The name originates from the idea that it is possible to perform an unlimited number of additions of different reagents to the sample and to perform multiple treatments (mixing, heating, cooling etc.) of the mixture at different stages of the sample preparation procedure.

The system was built using commercial modules some of which were modified for the purpose. The apparatus was controlled by a universal software operated from a personal computer. The equipment was therefore relatively inexpensive and very flexible.

This paper presents an analysis of primary amines to demonstrate the system. The analytical procedure includes pre-column coupling reaction of amines to fluorescent derivatives, followed by HPLC separation and fluorescence detection. For analysis of amino acids and amines there are many reagents of choice for derivatization. Among them are 1-dimethylaminophthalene-5-sulphonyl chloride (Dansyl-Cl), phenylisothiocyanate (PITC) or 9-fluorenylmethyl chloroformate (Fmoc). A long reaction time is needed when using Dansyl-Cl [3] and excess of reagent will give disturbing peaks [4]. The reaction described here is a modification of one described by Imai and Watanabe [5] using 4-Fluoro-7-nitrobenzofurazane (NBD-F) to form the fluorescent compound. The reagent itself is non-fluorescent and will form a stable fluorescence product with primary and secondary amines in a short time. The reaction produces only minor by-products so the evaluation of a chromatogram of NBD-F-derivatives is easy to handle and the identification is more certain.

### Materials and methods

### *Working principle of the system*

(1) Prepared samples in plastic tubes are introduced into a modified automatic sampler.
(2) A control program for a PC that executes a run list for the specific analysis is started. The number of sample tubes is noted in the running list and the procedure begins.
(3) The selector is turned to waste, and the dilutor is initialized. The injection syringe is forced into the top (empty) position and the rest of the contents of the syringe are drained to waste.
(4) A pre-set volume of buffer, reagent and sample is sucked up into the storage tube by the dilutor. The three solutions are separated from each other and from the transporting water in the tube by small segments of air which have been introduced via the waste channel.
(5) The different solutions are transported to the mixing cup. A thorough mixing then occurs by forcing air through the solution.
(6) The mixture is sucked back to the storage tube and then transported to the thermostatic module. After a pre-set heating time, during which the coupling reaction is completed, the dilutor forces the liquid back through the cooling compartment and once more into the mixing cup.
(7) The reaction is finally stopped by a pH change (an aliquot of acid is introduced). Mixing is performed by pressing air through the solution.

0142-0453/95 $10.00 © 1995 Taylor & Francis Ltd.

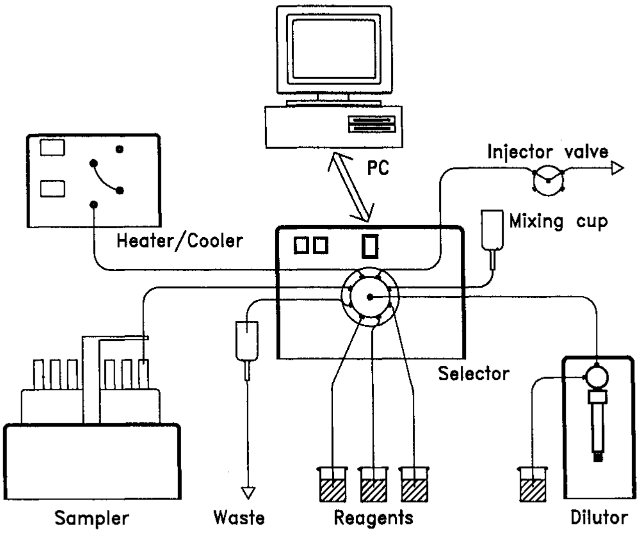

*Figure 1. The different parts of the automatic HPLC system.*

(8) The whole mixture is sucked back to the storage tube, the injector valve is put in the fill position and about half of the volume of the mixture is pressed through the injection loop.
(9) A signal is given to the injector valve that turns it to the inject position and another signal starts the integrator at the same moment. Separation is performed on the HPLC column.

The tube leading to the injector has a volume which is large enough to contain the second half of the mixture. This makes it possible to perform the derivatization of the next sample, during which time the double injection of the first sample takes place on the chromatograph. When the double analysis is finished, the next sample is already in the mixing cup and ready for its injection. This way of working means that a very short time is necessary for analysis.

*Details of the apparatus*

The apparatus consists of the following items (see figure 1):

(1) Auto sampler (Varian, Auto sampler 8000, storage module, modified for automatic control from a PC).
(2) Computer (Turbo μnitron micro PC 2900ST, IBM compatible) with two communication ports RS232C (COM1 and COM2) and an option card with 24 I/O pins (PC-36A, Boston Technology).
(3) Selector valve (Analys Modul AB Sweden).
(4) Dilutor with a 1000 μl syringe (Microlab M, Hamilton Bonaduz AG, Bonaduz, Switzerland).
(5) Thermostatic heater/cooler module (max. 95°C and min 10°C, Analys Modul AB, Sweden).
(6) Injection valve (Rheodyne 5701 Pneumatic Actuator, 7010 Sample injection valve and 7163 Solenoid valve kit. Rheodyne Inc., Cotati, CA, USA).

Flow speed and stroke volume for the dilutor (on COM2), as well as the port number on the selector valve (on COM1), was controlled from the run list. The flowing streams from the dilutor were directed

| Device | Port | Command to device | Users comments |
|---|---|---|---|
| SEL | COM1 | POS 2.0 | Valve port to mixing cup |
| HAM_DIL | COM2 | P 50 | Aspirate 50 μ air |
| SEL | COM1 | POS 7.0 | Valve port to sampler |
| HAM_DIL | COM2 | P 400 | Aspirate 400 μl of sample |
| SEL | COM1 | POS 6.0 | Valve port to waste |
| HAM_DIL | COM2 | D 450 | Dispense 450 μl to waste |
| HAM_DIL | COM2 | I | Dilutor port to wash cup |
| HAM_DIL | COM2 | PICKUP_SPEED 5 | Set dilutor aspir. speed |
| HAM_DIL | COM2 | P 1000 | Aspirate 1000 μl water |
| HAM_DIL | COM2 | O | Dilutor port to normal |
| HAM_DIL | COM2 | D 1000 | Wash waste tube |
| HAM_DIL | COM2 | PICKUP_SPEED 20 | Set dilutor aspir. speed |
| SEL | COM1 | POS 2.0 | Valve port to mixing cup |
| HAM_DIL | COM2 | P 25 | Aspirate 25 μl air |
| SEL | COM1 | POS 5.0 | Valve port to buffer cup |
| HAM_DIL | COM2 | P 100 | Aspirate 100 μl buffer |
| SEL | COM1 | POS 2.0 | Valve port to mixing cup |
| HAM_DIL | COM2 | P 25 | Aspirate 25 ul air |
| SEL | COM1 | POS 7.0 | Valve port to sampler |
| HAM_DIL | COM2 | P 100 | Aspirate 100 μl sample |
| SEL | COM1 | POS 2.0 | Valve port to mixing cup |
| HAM_DIL | COM2 | DISP_SPEED 15 | Set dilutor disp. speed |
| HAM_DIL | COM2 | D 100 | Sample to mixing cup |
| SEL | COM1 | POS 3.0 | Valve port to reag. cup 1 |
| HAM_DIL | COM2 | P 200 | Pick up 200 μl reagent 1 |
| SEL | COM1 | POS 2.0 | Valve port to mixing cup |
| HAM_DIL | COM2 | D 350 | Reagent to mixing cup |
| SEL | COM1 | POS 6.0 | Valve port to waste cup |
| HAM_DIL | COM2 | P 800 | Aspirate water/lair |
| SEL | COM1 | POS 2.0 | Valve port to mixing cup |
| HAM_DIL | COM2 | D 800 | Mix through disp. air |

*Figure 2. Run parameters as displayed on the PC screen. The operator's changes are typed in the column called 'Command to device'. The right-hand column shows the step by step execution of the automatic run list.*

in different directions via the selector valve with its eight channels. All connecting tubings were of Teflon with I.D. 1.35 mm, except for the tubings from the auto sampler and to the injector valve. Their I.D.s were 0·5 mm. The control program and all modifications of instruments were made by Analys Modul Sweden AB. The software was a universal controlling program, EASYLAB, from Analys Modul Sweden AB.

*Instrumentation for HPLC*

The pump was an LKB 2150 HPLC pump (Pharmacia Biotech AB, Sollentuna, Sweden) with a Resolve 5 μ column (Spherical $C_{18}$, 39 × 150 mm, Waters, Millipore Corp., Milford, MA, USA) with a pre-column (Resolve, Guard-Pak, Waters). Fifty μl of sample was injected from a loop. The eluent solution was methanol-water (55:45), with a constant flow of 1 ml/min. Detection was made by fluorescence at excitation wavelength 480 nm and emission wavelength 530 nm (Fluorescence HPLC Monitor, RF-535, Shimadzu Co, Kyoto, Japan). Calculations and integration of chromatograms were made by an ELDS 900 lab data system for chromatography (Chromatography Systems AB, Stenhamra, Sweden).

*Derivatization reaction*

From the absorption solution (HCl, 15 mmol/l) in the diffusive sampler, 1500 μl was transferred to a sample vial in the auto injector. From that 100 μl was taken for the reaction. The sample was buffered to alkaline pH with 100 μl borate/HCl buffer (0·1 mol/l, pH 8·8) and mixed with 200 μl NBD-F (2·7 mmol/l in ethanol; freshly prepared). The mixture was heated for 5 min at 55°C and cooled for 5 s at 15°C. Then 400 μl HCl (0·5 mmol/l) was added to stop the reaction.

*Table 1. CVs (%) for double injections of two different concentrations from five vials containing the same mixture of six primary amines. The quotient between each double injection and its corresponding CVs is shown, as well as arithmetic means of the CVs.*

| | 500 pmoles of amine | | 100 pmoles of amine | |
|---|---|---|---|---|
| | Peak area | Peak height | Peak area | Peak height |
| Injection I | 1·51 | 2·10 | 5·39 | 5·00 |
| Injection II | 2·00 | 2·47 | 5·84 | 5·35 |
| Inj I/Inj II (CV) | 1·01 (0·96) | 1·04 (0·36) | 1·00 (2·95) | 1·01 (0·95) |

*Chemicals*

(1) Methylamine hydrochloride (purum), ethylamine hydrochloride (puriss), propylamine (puriss) (Fluka AG, Buchs, Switzerland).
(2) N-butylamine, iso-butylamine, sec-butylamine, ampoules for analytical standards (PolyScience Corp., Niles, IL, USA).
(3) NBD-F, ampoules 5 mg. (Sigma Chemical Co, St Louis, MO, USA).
(4) Boric acid, crist. (p.a.) and methanol (p.a.) (Merck, Darmstadt, Germany).

*Separation test mixtures*

A mixture of six primary amines was tested at two different concentrations. Double aliquots were taken from each sample vial; 100 and 500 pmol of each amine respectively were injected.

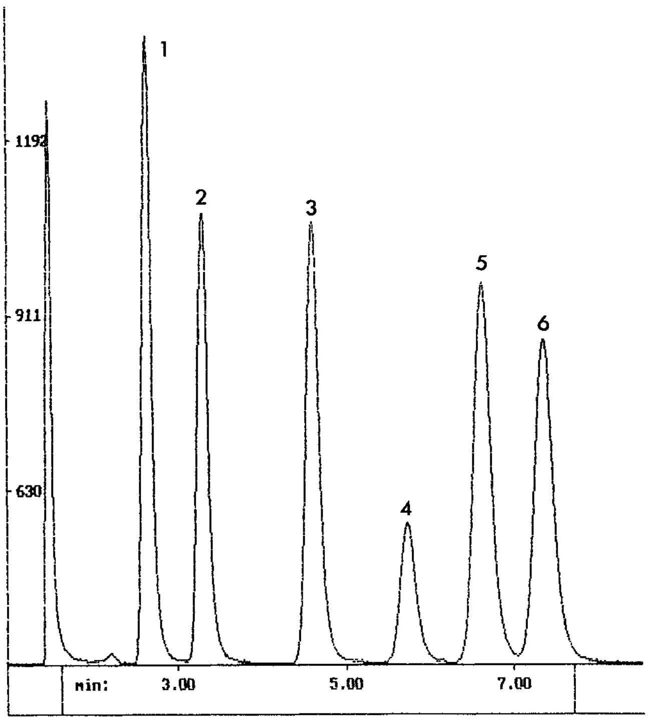

*Figure 3. Separation of six primary amines after auto derivatization and injection. 1 methyl-, 2 ethyl-, 3 propyl-, 4 sec-butyl-, 5 iso-butyl-, 6 n-butylamine (500 pmoles of each).*

### Results and discussion

Figure 3 shows a chromatogram with the separation of six primary amines. The areas as well as the peak heights, from the double injections are better correlated for those peaks measured at higher concentrations. There is no statistical significant difference between double injections of samples (Student's t-test, 1% level). Tables 2 and 3 show that bigger peaks give better analytical precision. There is also a slightly better precision if the whole reaction is done manually. This is the case for short series of samples. The advantage of automation is that a long series with many samples can be analysed in a uniform way.

When 100 pmoles of each amine was injected, the mean peak area for all six amines was 1·14 (SD 0·05, CV 4·33). For peak heights the corresponding mean was 1·11 (SD 0·04, CV 3·71). (All other parameters are as in table 4.)

*Table 2. Comparison of precision between automatic and manual analysis of fluorescent derivatives from six primary amines (CV%). 500 pmoles of amine were injected in five to seven consecutive injections.*

| | Auto injection | | Manual injection | |
|---|---|---|---|---|
| | Peak area | Peak height | Peak area | Peak height |
| Methylamine | 1·20 | 1·75 | 0·81 | 0·65 |
| Ethyl- | 1·56 | 2·15 | 0·91 | 0·39 |
| Propyl- | 2·25 | 1·99 | 1·06 | 0·51 |
| s-butyl- | 0·94 | 2·14 | 1·44 | 0·42 |
| iso-butyl- | 1·02 | 1·40 | 0·82 | 0·65 |
| n-butyl- | 1·64 | 1·53 | 1·04 | 0·42 |
| Mean | 1·44 | 1·83 | 1·01 | 0·51 |
| SD | 0·49 | 0·32 | 0·23 | 0·12 |
| CV | 33·9 | 17·5 | 23·1 | 23·3 |

*Table 3. Conditions as table 2 but with 100 pmoles of amine.*

| | Auto injection | | Manual injection | |
|---|---|---|---|---|
| | Peak area | Peak height | Peak area | Peak height |
| Mean | 5·29 | 5·09 | 4·35 | 2·97 |
| SD | 1·21 | 0·81 | 2·03 | 3·45 |
| CV | 22·9 | 15·9 | 46·7 | 116·1 |

*Table 4. Quantitative comparison between manual and automatic injections. Mean of quotient manual/automatic shown for five consecutive injections of six different amines (500 pmoles of each) in a mixture.*

| | Peak area | | | Peak height | | |
|---|---|---|---|---|---|---|
| | Mean | SD | CV | Mean | SD | CV |
| Methylamine | 1·08 | 0·01 | 0·82 | 1·06 | 0·02 | 1·52 |
| Ethyl- | 1·07 | 0·01 | 1·07 | 1·05 | 0·02 | 2·12 |
| Propyl- | 1·07 | 0·03 | 3·01 | 1·07 | 0·02 | 1·81 |
| sec-butyl- | 1·08 | 0·02 | 1·75 | 1·07 | 0·02 | 2·15 |
| iso-butyl- | 1·12 | 0·01 | 0·76 | 1·10 | 0·00 | 0·34 |
| n-butyl- | 1·15 | 0·03 | 2·27 | 1·14 | 0·02 | 1·46 |
| All six amines | 1·09 | 0·03 | 2·90 | 1·08 | 0·03 | 2·98 |

*Table 5. Retention times relative to methylamine (= 1·00) for six primary amines. Double samples in five consecutive injections.*

| | Injection I | | | Injection II | | |
|---|---|---|---|---|---|---|
| | Mean | SD | CV | Mean | SD | CV |
| Ethylamine | 1·26 | 0·01 | 0·64 | 1·27 | 0·00 | 0·23 |
| Propyl- | 1·79 | 0·04 | 2·31 | 1·79 | 0·01 | 0·50 |
| sec-butyl- | 2·25 | 0·05 | 2·39 | 2·27 | 0·03 | 1·36 |
| iso-butyl- | 2·61 | 0·06 | 2·43 | 2·64 | 0·04 | 1·40 |
| n-butyl- | 2·90 | 0·07 | 2·46 | 2·93 | 0·04 | 1·37 |

Manual injections give a slight increased fluorescence intensity compared to auto injection. This is probably due to dilution in tubing system or incomplete mixing at automation. However, correspondence in repeatability is of more importance than absolute intensity.

The relative retention times do not change between the injections (see table 5). The fluorescence relative methylamine (= 1·00) was at the same magnitude for both concentrations investigated. It was between 0·81 and 1·14 for all amines, except sec-butylamine (0·39).

### Conclusions

The automatic system described here is easy to handle and works with good precision. It is flexible and cost effective. The different separation parameters in the program run protocol are easy to change and the system can easily be adapted to any analytical method. However, the washing procedure between different samples must be checked carefully to avoid carry-over in the transport tubes and mixing vial; volumes and flow speed have to be set accordingly. Standard solutions of known concentration must be placed in the auto sampler at fixed intervals to ensure good analytical precision.

### Acknowledgement

This work was supported financially by the Swedish Work Environment Fund.

### References

1. Felder, R. A., *Drugs and the Pharmaceutical Sciences*, **47** (1991), 185.

2. Ruzicka, J. and Hansen, E. H., in *Flow Injection Analysis* (John Wiley & Sons Inc., New York, 1988).

3. Simmaco, M., De Biase, D., Barra, D. and Bossa, F., *Journal of Chromatography*, **504** (1990), 129.

4. Gustavsson, B. and Betner, I., *Journal of Chromatography*, **507** (1990), 67.

5. Imai, K. and Watanabe, Y., *Analytica Chimica Acta*, **130** (1981), 377.

